# Preparation of Silver/Chitosan Nanofluids Using Selected Plant Extracts: Characterization and Antimicrobial Studies against Gram-Positive and Gram-Negative Bacteria

**DOI:** 10.3390/ma13071629

**Published:** 2020-04-01

**Authors:** Saviour A. Umoren, Moses M. Solomon, Alexis Nzila, Ime B. Obot

**Affiliations:** 1 Center of Research Excellence in Corrosion, Research Institute, King Fahd University of Petroleum and Minerals, Dhahran 31261, Saudi Arabia; moses.solomon@kfupm.edu.sa (M.M.S.); obot@kfupm.edu.sa (I.B.O.); 2 Department of Life Sciences, College of Science, King Fahd University of Petroleum and Minerals, Dhahran 31261, Saudi Arabia; alexisnzila@kfupm.edu.sa

**Keywords:** *Phoenix dactylifera*, *Rumex vesicarius*, nanofluid, chitosan, silver nanoparticles, antimicrobial effect

## Abstract

Chitosan/silver nanofluids were prepared using *Phoenix dactylifera* (DPLE) or *Rumex vesicarius* (HEL) extracts as the reducing agent, characterized using Fourier-transform infrared spectroscopy (FTIR), ultraviolet–visible (UV-vis), X-ray diffraction (XRD), and transmission electron microscope (TEM). The antimicrobial effect of the nanofluids against Gram positive, *Bacillus licheniformis*, *Staphylococcus haemolyticus*, *Bacillus cereus*, and *Micrococcus luteus*, and Gram-negative *Pseudomonas aeruginosa*, *Pseudomonas citronellolis*, and *Escherichia coli* bacteria has been studied. The nanoparticles were polydispersed in the chitosan matrix and are highly stable. The zeta potential of the silver nanoparticles in DPLE- and HEL-mediated composites is +46 mV and +56 mV, respectively. The FTIR results reveal that the free carboxylate groups in the plant biomaterial took part in stabilization process. HEL is a stronger reducing agent than DPLE and nanoparticles generated with HEL are smaller (8.0–36 nm) than those produced with DPLE (10–43 nm). DPLE- and HEL-mediated composites effectively inhibit the growth of the studied bacteria but HEL-mediated composite exhibited higher effect. The higher antimicrobial activity of HEL-mediated composite is linked to the smaller nanoparticles. The foregoing results indicate that HEL extract can be used in the green production of potential antimicrobial chitosan/silver nanofluids for biomedical and packaging applications.

## 1. Introduction

Microorganisms play very vital roles in many life-sustaining processes. Nevertheless, some are pathogenic causing illness and even death. Although many antibiotics are available for the management of bacterial infections, the emerging infectious diseases and the resistance of bacteria strains to antibiotics at unprecedented rate have make the search of new antimicrobials a necessity. In addition, the environmental concern over some antibiotics [1] also informed the recent interest in searching for safe and natural antibiotics replacement [1,2].

Time immemorial, the antimicrobial characteristic of silver ions had been known [3]. It is on record that the ancient Greek used silver for stomach pains or wound healing [4]. Recent findings have shown that, silver nanoparticles (AgNPs) because of their high specific surface area and high fraction of surface atoms have higher antimicrobial property than the bulk silver metal [5,6,7]. This is because of the minute size and the appreciable surface: the volume ratio of the nanoparticles promotes the interaction of the nanoparticles with microbes [8,9]. The wide range of applications of metal nanoparticles have been extensively reported [8,9]. It had been demonstrated that silver nanoparticles/polymer composites exhibit enhanced antimicrobial activity [10,11,12,13], and hence can have application in medical treatments.

**Figure 1:**
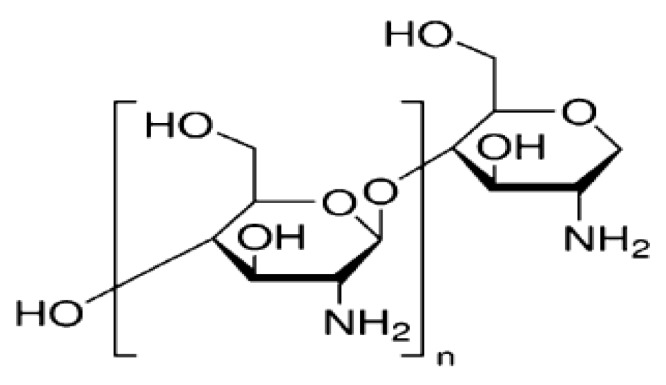
Molecular structure of chitosan.

Chitosan (Figure 1) is among the commonly found natural polymers [14,15]. It has diverse applications in the medical, food, and pharmaceutical fields because of the antibacterial, nontoxicity, good biodegradability, and biocompatibility properties [16,17]. Its antibacterial effect is due to the presence of protonated groups in the polymer backbone and the ionic interplays between the charged groups and the bacteria wall constituents [18]. Consequently, the peptidoglycans in microorganism wall is hydrolyzed provoking the leaking of intracellular electrolytes and the death of the microorganism [18].

The charges on chitosan backbone can be created by protonation of the –NH_2_ groups in acid solution or by structural modification (i.e., methylation, sulfonation, etc.) [1,18]. Reports have shown that structural modification improves resistance property of chitosan to bacteria. Goy et al. [18] documented a superior inhibition effect of *N, N, N*-trimethylchitosan against Gram-positive and Gram-negative bacterium strains relative to neat chitosan. *P. aeruginosa* biofilm formation and adhesion was reported by Liu et al. [1] to be inhibited by sulfonated chitosan and chitosan hydrochloride. N-quaternary ammonium-O-sulfo-betaine-chitosan had equally been reported to show improved bacteria resistance effect and water solubility [19].

It is expected that, AgNPs/chitosan nanofluid will exhibit better antimicrobial activity than chitosan or AgNPs alone. Therefore, in this communication, AgNPs/chitosan nanofluids synthesized using *Phoenix dactylifera* or *Rumex vesicarius* leaves extracts as cost-effective reducing agent are utilized as antimicrobial agent against Gram-positive (*Bacillus licheniformis*, *Staphylococcus haemolyticus*, *Bacillus cereus*, and *Micrococcus luteus*) and Gram-negative (*Pseudomonas aeruginosa*, *Pseudomonas citronellolis*, and *Escherichia coli*) bacteria. Two reducing agents (*Phoenix dactylifera* and *Rumex vesicarius*) were used in the synthesis process in order to establish the role of the reducing agent on the nanoparticles size and in extension antimicrobial property. *Phoenix dactylifera* and *Rumex vesicarius* are readily available in the Middle East hence a cost-effective source.

The biosynthesis of metals nanoparticles using plant extracts is seen as an alternative technique to the chemical, physical, and the microbial techniques [20] because it is facial, green-compliance, inexpensive, and suitable for large-scale production [21,22]. Beside the aforementioned advantages, metals nanoparticles produced using plant extracts as the reducing and stabilizing agents are found to be very stable and safe for packaging and human therapeutic applications [20,22,23,24]. For instance, Zayed et al. [20] reported −16 mV and −13 mV as the zeta potentials for silver and gold nanoparticles, respectively produced using *Pimpinella anisum* seeds extract as the reducing and stabilizing agent. These values are indicative of highly stable nanoparticles. The synthesized silver and gold nanoparticles when tested for their free radical scavenging activity against 1,1-diphenyl-2-picryl-hydrazyl and antimicrobial activity against *E. coli*, *S. aureus*, *Aspergillus flavus* and *Candida albicans* showed high antioxidant and antimicrobial activities. In a similar study, Jha et al. [25] recently demonstrated that highly stable silver nanoparticles suitable for therapeutic applications can be synthesized using the extracts of *Citrus maxima* plant. Several of such reports can be found in the literature [7,21,22,23,26,27].

To the best of our knowledge, there is no report on the synthesis of AgNPs/chitosan nanofluid using *Phoenix dactylifera* or *Rumex vesicarius* leaves as the biomaterials. There term ‘nanofluid’ is adopted to reflect the liquid state of the composite.

## 2. Materials and Methods

### 2.1. Materials

Chitosan (Mol. wt.: 50,000–190,000 Da, degree of deactylation: 75–85%, viscosity: 20–30 cP), silver nitrate (≥99.0%), and acetic acid (≥99.0%) were Merck products and were used without further purification. Fresh *Phoenix dactylifera* (DPLE) and *Rumex vesicarius* (HEL), leaves were collected at the King Fahd University of Petroleum and Minerals (KFUPM) campus and validated by a botanist, Dr. Jacob Thomas from King Saud University (KSU), Riyadh, Saudi Arabia. The plant specimens have been deposited in the herbarium with the voucher numbers KSU No. 22638 and KSU No. 20872 for *Phoenix dactylifera* and *Rumex vesicarius*, respectively.

Seven bacteria strains isolated from the petroleum-contaminated shorelines of the Arabian Gulf of Saudi Arabia were provided by the Department of Life Sciences, King Fahd University of Petroleum and Minerals, Saudi Arabia. The Gram-negative strains include *Pseudomonas aeruginosa* (GI482716237, gene accession number), *Pseudomonas citronellolis* (KT894554), and *Escherichia coli* (ATCC 25992, reference on ATCC global resource). The Gram-positive strains consist of *Bacillus licheniformis* (KF609498), *Staphylococcus haemolytic* (MN388897), *Bacillus cereus* (MN888756), and *Micrococcus luteus* (MN888755). These gene accession numbers can be found in National Center of Biotechnology Institute (NCBI) [28].

### 2.2. Plant Leaves Extraction

The DPLE and HEL leaves were thoroughly washed, dried in the sun for 14 days, and grounded into powder form. For extraction, 5.0 g of the respective leaves powder was boiled in 500 mL distilled water under constant stirring at 200 rpm for 3 h. Thereafter, it was left at room temperature to cool, and then filtered making use of Whatman^®^ (United States reference) Grade 1 filter papers (Merck). The filtrate was preserved in a refrigerator.

### 2.3. Preparation of AgNPs/Chitosan Nanofluids

The preparation procedure involves series of steps. Firstly, 2.0 g of chitosan was added to 100 mL of 0.1 M CH_3_COOH acid solution and stirred. Secondly, 0.02 g of AgNO_3_ dissolved in 5 mL of distilled water was introduced to the polymer solution obtained in the first step. The chitosan- AgNO_3_ solution was stirred at 150 rpm for 3 h. Thirdly, 5 mL of DPLE or HEL extract was added to the chitosan-AgNO_3_. Fourthly, the DPLE or HEL extract- chitosan-AgNO_3_ solution was left at room temperature under constant stirring for 24 h. The AgNPs/chitosan nanofluid synthesized using DPLE leaves extract is herein referred to as DPLE-mediated composite while the one prepared using HEL leaves extract is designated as HEL-mediated composite.

### 2.4. Characterization

The nanofluids, the DPLE and HEL extracts, and chitosan were characterized using a Fourier-transform infrared spectroscopy (FTIR) spectrophotometer (Nicolet iS5, Thermo Scientific model, United States) over the range 4000 to 400 cm^−1^. 

The ultraviolet-visible (UV-vis) spectra of the developed DPLE- and HEL-mediated composites were obtained using a JASCO770-UV–Vis (Tokyo, Japan) spectrophotometer (200–650 nm). A scan rate of 200 nm·min^−1^ was used and was operated at a resolution of 1 nm.

For X-ray diffraction (XRD) characterization, the DPLE- and HEL-mediated composite colloidal solutions were centrifuged at 10,230 rpm for 25 min. The solid residues obtained from the process were washed thrice with ultrapure water. The residues were re-dissolved in absolute ethanol, evaporated to dryness at 50 °C, and the powder sample submitted for XRD analysis. A Rigaku MiniFlex X-ray diffractometer (Tokyo, Japan) was used.

The transmission electron microscope (TEM), JEOL instrument JEM-2100F model (Tokyo, Japan) was used to characterize the morphology and size of AgNPs in the composite. To achieve this, a drop of the colloidal DPLE- or HEL-mediated composite was loaded on a carbon-coated Cu sample holder and air-dried at normal temperature. The accelerating voltage used was 200 kV.

Finally, the zeta potential and the polydispersity index (PDI) of AgNPs in the composites were determined using a Malvern Instrument, Zetasizer ver. 7.12 (United Kingdom).

### 2.5. Bacteria Cultures

Bacterial cultures were revived from cryopreserved bacterial samples, by culture in rich Luria broth medium (LB), at 37 °C, 120 rpm, for 3 days. These revived bacterial cells were then used in subsequent experiments. For growth inhibition assessment in liquid culture (Section 2.7), bacteria were cultured in LB medium, in the presence of inhibitors (nanoparticles and antibacterial agents) at 37 °C, 120 rpm for one day, and their growth monitored by visual observation of the turbidity and by quantification [7]. In relation with solid agar plate cultures for “cup-plating”, an incubation period of 12 h was used (Section 2.6), while for bacterial counting, the incubation was one day (Section 2.7). All experiments were carried out in duplicate and the mean value alongside the standard deviation are presented.

### 2.6. Antibacterial Activity by ‘cup-plating’ 

The ‘cup and plating’ technique was deployed in the assessment of the bacteria inhibiting efficacy of AgNPs/chitosan nanofluids [7,29]. The technique, which is related to the Disk-diffusion test, consists of a preparation of a solid agar plate in rich medium, on which 100 µL of a bacterium culture containing 10^6^ CFU/mL was streaked. Thereafter, and a central hole of a depth of 5 mm in diameter and 2 mm height was created, in which 100 µL of the nanoparticles was added. These agents diffused from the central point of the hole to the rest of the plate, and as this diffusion took place, the agents would inhibit the growth of bacteria present on the plate, creating an inhibition zone (disc diffusion). This inhibition zone was assessed after 12 h incubation period. The higher the activity of the antibacterial agents, the higher inhibition zone, and these zones were quantified by measuring their diameters. This approach of “cup-plating” had been previously reported [29].

### 2.7. Assessment of the Minimum Inhibitory Concentration (MIC) and Minimum Bactericidal Concentration (MBC)

In liquid culture media, bacterial growth, in the presence of different concentration (0.001–10%) of AgNPs/chitosan nanofluids, was assessed visually, by monitoring the culture turbidity, which is indicative of the presence of both living and dead bacteria [7,29]. From the culture, MIC was assessed and it indicates the bacteriostatic effect or the lowest antimicrobial agent dosage at which no turbidity is observed.

In order to assess the viable bacteria only, the liquid culture media containing different concentrations (0.001–10%) of AgNPs/chitosan nanofluids were transferred to solid plates according to the following protocol. About 100 µL of culture was spread on agar LB plate and incubated at 37 °C for 24 h. In the agar plate, only viable bacteria can grow and generate colony-forming units (CFUs). Since in the initial liquid medium, bacterial concentration was high, an initial dilution of the culture (by factor of ten for five dilutions) was carried out prior to the transfer in solid plate. Viable bacteria were counted as CFUs, and the least dosage of AgNPs/chitosan nanofluids that retards bacterial growth in the solid plate is defined as minimum bactericidal concentration (MBC). In these experiments, negative control (NC) consisted of culture medium devoid of bacteria and antimicrobial agent. The essence of NC was to ascertain the absence of bacterial contamination of the culture media. On the other hand, positive control contained bacterial cultures without inhibitors and was employed to assess the maximum growth of bacteria.

## 3. Results and Discussion

### 3.1. Physical Appearance of Synthesized AgNPs/Chitosan Nanofluids

**Figure 2:**
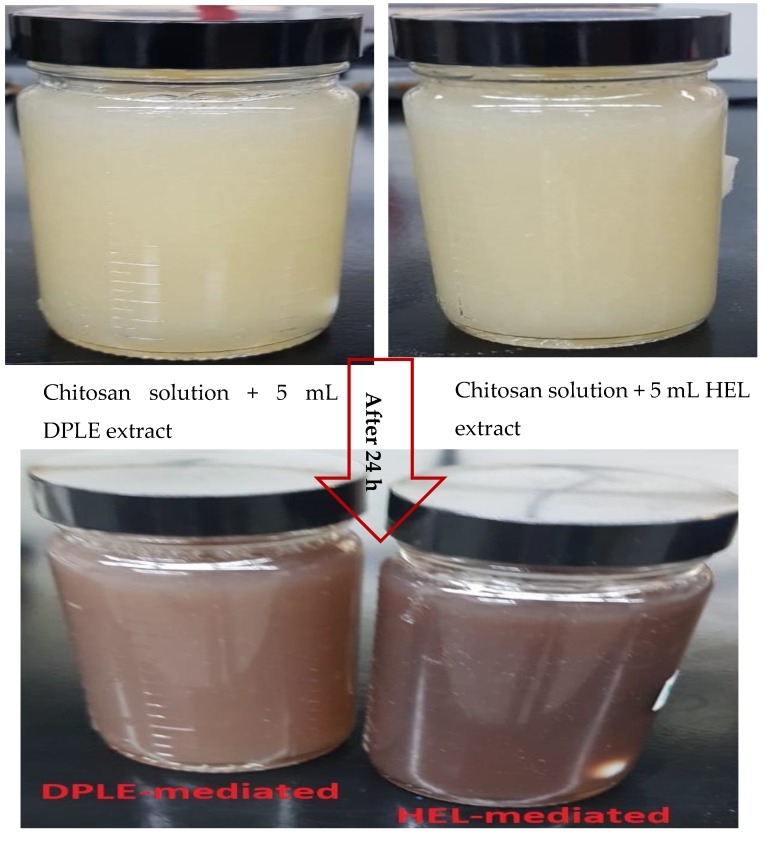
Digital photographs of color change of DPLE- and HEL-mediated composites.

Upon addition of DPLE or HEL extract to chitosan-AgNO_3_ solution, a yellowish color solution was obtained (Figure 2). By allowing the yellow color solution to stand at room temperature under constant stirring for 24 h, the solution was decolorized from yellowish to dark-brown color (Figure 2). The change in color signaled the formation of silver nanoparticles [30]. An addition of sodium chloride solution to a small portion of the dark-brown nanofluid failed to form white precipitate expected from the reaction of sodium chloride and silver ions. This indicated that the Ag^+^ ions was converted completely to Ag^0^ [30].

### 3.2. FTIR Studies

**Figure 3:**
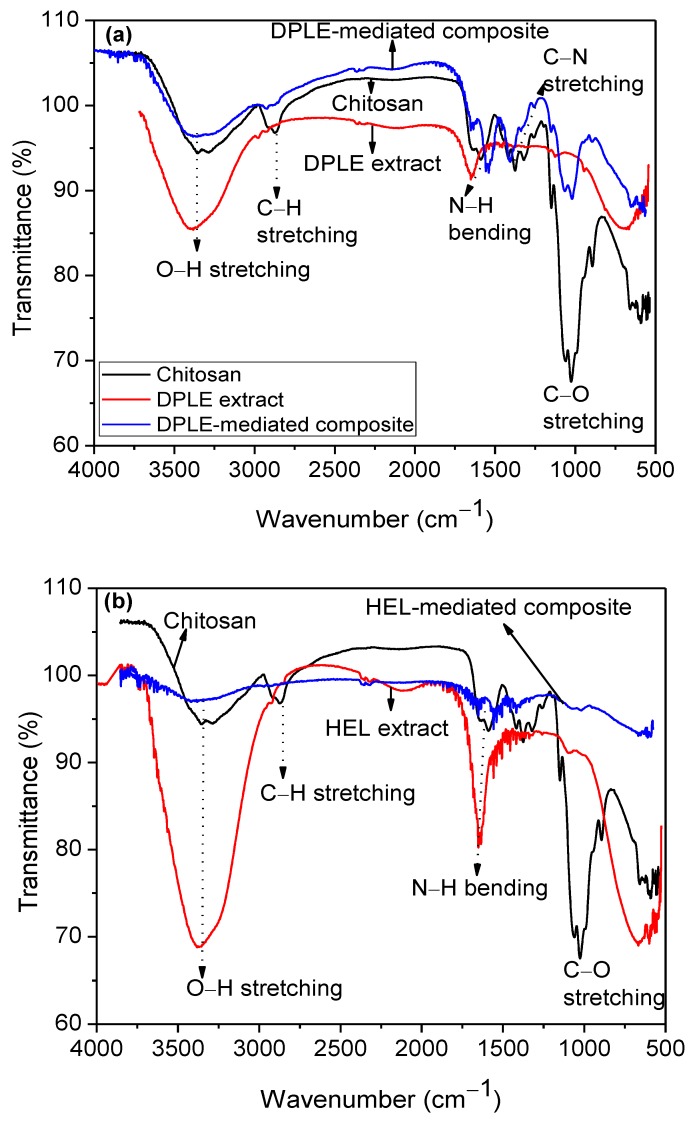
FTIR spectra of (**a**) chitosan, DPLE leaves extract, and DPLE-mediated composite; (**b**) chitosan, HEL leaves extract, and HEL-mediated composite.

Figure 3 presents the attenuated total reflectance-infrared (ATR-IR) spectra of (a) chitosan, DPLE leaves extract, and DPLE-mediated composite and (b) chitosan, HEL leaves extract, and HEL-mediated composite. The IR spectrum of the neat chitosan show distinct peaks at 3358.21 cm^−1^, 2873.75 cm^−1^, 1589.35 cm^−1^, 1375.48 cm^−1^, 1149.66 cm^−1^, 1026.14 cm^−1^, and 893.56 cm^−1^. Distinct peaks are found in the DPLE leaves extract at 3383.10 cm^−1^, 1652.17 cm^−1^, 1557.78 cm^−1^, 1539.56 cm^−1^, 1405.49 cm^−1^, 1151.06 cm^−1^, 1019.85 cm^−1^, and 653.19 cm^−1^. For HEL extract, bands are seen at 3382.41 cm^−1^, 1732.67 cm^−1^, 1651.87 cm^−1^, and 667.35 cm^−1^. The DPLE-mediated composite exhibits peaks in the IR spectrum at 3383.10 cm^−1^, 1652.17 cm^−1^, 1557.78 cm^−1^, 1539.56 cm^−1^, 1405.49 cm^−1^, 1151.06 cm^−1^, 1019.85 cm^−1^, and 653.19 cm^−1^. Similarly, the HEL-mediated composite shows characteristic peaks at 3418.36 cm^−1^, 2356.67 cm^−1^, 1574.98 cm^−1^, 1417.94 cm^−1^, 1019.31 cm^−1^, and 608.29 cm^−1^. The band around 3350–3390 cm^−1^ in the spectra is typical of the O–H stretching of polyphenols [31]. The peak in the region of 2870 cm^−1^ is assigned to the C–H stretching [31,32]. Expectedly, the N–H and C–N peaks are observed at around 1600 and 1500 cm^−1^, respectively. The intense band at around 1100 cm^−1^ in the spectra is consistent with the vibration of C–O [31,32].

A comparison of the FTIR spectra of DPLE-mediated and HEL-mediated composites with those of chitosan and the plant extracts reveals that the intensity of the O–H and C–O vibration bands significantly diminished in the composites spectra. There is also a slight shift in the position of the bands in the composite spectra relative to those of the plant extracts. This suggests the involvement of the plant phytochemicals in the reactions that converted Ag^+^ ions to atomic Ag. It was reported [33,34,35] that DPLE and HEL leaves are rich in phytochemicals (namely, tannins, flavonoids, saponins, alkaloids, steroids, phenols, terpenoids, carbohydrates, and amino acids). These phytochemicals have been claimed to be responsible for the reduction of the AgNO_3_ to AgNPs [6,7,23]. Additionally, the peaks observed at 3418.36 cm^−1^ in the HEL-mediated composite spectrum and the one at 1652.17 cm^−1^ in the DPLE-mediated composite spectrum indicate the binding of proteins, saccharides, and nitrogenous compounds on the surface of the AgNPs [27]. Such binding brings about the stability of the AgNPs [36,37,38]. As it is known, nanoparticles can be stabilized through free –NH_2_ groups or RCOO^–^ ions of protein’s amino acid residue [36,37,38]. These two cases can be differentiated by considering whether or not there is C=O vibration band at approximately 1700 cm^−1^. In a typical scenario of that biosynthesized Au nanoparticles were stabilized via free –NH_2_ groups, C=O vibration peak was noted at 1714 cm^−1^ [39]. In the present study (Figure 3), the C=O stretching band is not observed in the spectra of the biosynthesized composites, as such the stabilization of the AgNPs is considered to be through the free carboxylate group.

By comparing the FTIR spectrum of DPLE-mediated composite (Figure 3a) with the HEL-mediated composite spectrum (Figure 3b), it is observed that HEL-mediated composite spectrum exhibits less intense peaks compare to those in the DPLE-mediated composite spectrum. In fact, the C–H and C–N stretching bands are near absent in the HEL-mediated composite spectrum. This seems to suggest a higher reduction ability by the HEL extract than the DPLE extract. This assertion is also supported by the darker coloration of the HEL-mediated composite compare to the DPLE-mediated composite (Figure 2). In a study on the chemical composition of HEL leaves conducted by Alfawaz (2006), it was reported that, the range of organic acids was 277–307 mg/100 g for citric, 5530–5620 mg/100 g for malic, and 2840–3260 mg/100 g for oxalic acid. The protein content was put at 17.1–20.1 g/100 g. For DPLE leaves, Mohamed et al. [40] documented the organic composition to be in the range of 35.82–99.34 mg gallic acid equivalent/100 g and 1.74–3.39 mg catechin equivalent/100 g. Going by these reports, the HEL leaves are richer in organic phytochemicals than the DPLE leaves. This could make the HEL leaves extract a better reducing agent than the DPLE leaves extract as evidenced in the darker coloration of the HEL-mediated composite relative to the DPLE-mediated composite.

### 3.3. UV-vis Studies

**Figure 4:**
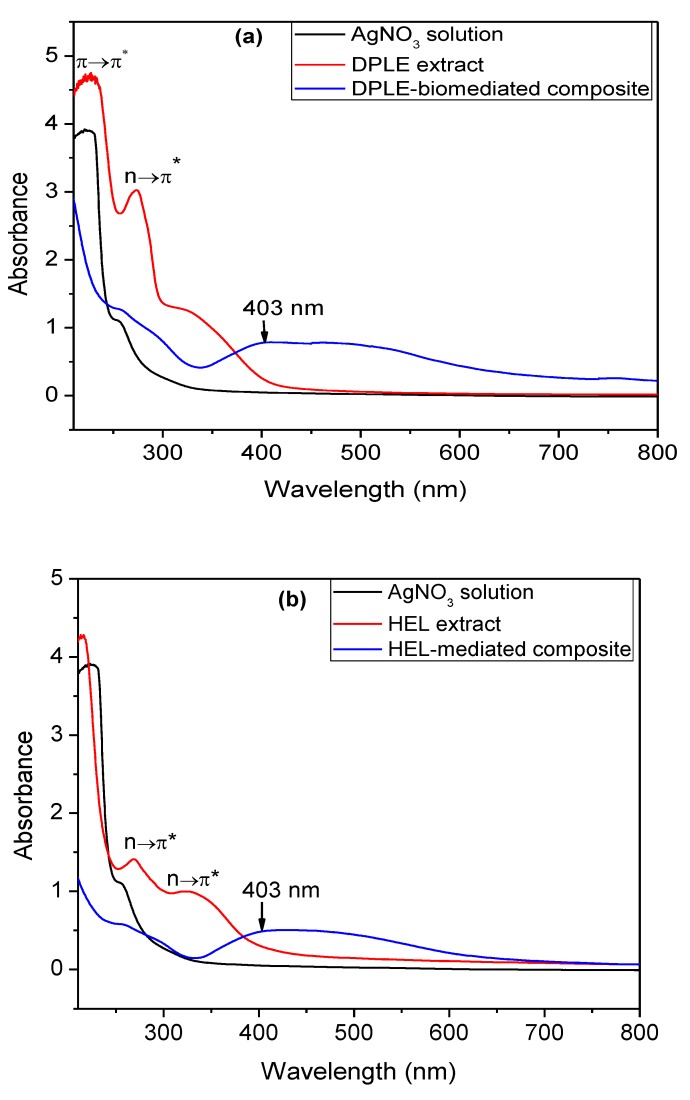
Comparative UV-vis spectra of (**a**) AgNO_3_ solution, DPLE leaves extract, and DPLE-mediated composite; (**b**) AgNO_3_ solution, HEL leaves extract, and HEL-mediated composite.

One of the reliable techniques for the confirmation of silver nanoparticles formation is the UV-vis technique. This is because of the extraordinary efficiency with which AgNPs absorb and scatter light [41]. This unique interaction takes place because of the so-called surface plasmon resonance effect [41]. Generally, AgNPs absorb light and give signal in the visible region at 380–450 nm [42]. The peak position is, however, influenced by the particle size, shape, and the local refractive index [41]. Small sized and spherically shaped nanoparticles absorb and give peak near 400 nm while larger nanospheres and/or polydispersed nanoparticles produced broaden peak that shifts toward longer wavelengths—red shifting [30,41]. The UV-vis spectra obtained for the DPLE- and HEL-mediated composites are shown in Figure 4. For comparison purpose, the UV-vis spectra of the extract alone and the AgNO_3_ + chitosan solution are also presented in Figure 4. It is apparent in the figure that the plant extracts successfully reduced silver ions to silver nanoparticles. As clearly seen in the figure, the unique silver resonance transition peak is at 403 nm in the DPLE- and HEL-mediated composites spectra. This peak is absent in the plant extracts and AgNO_3_ + chitosan spectra. However, the silver resonance transition peak is broad. As stated earlier, this is a characteristic of larger sized nanoparticles [41] and/or polydispersed nanoparticles [30].

### 3.4. TEM Studies

**Figure 5:**
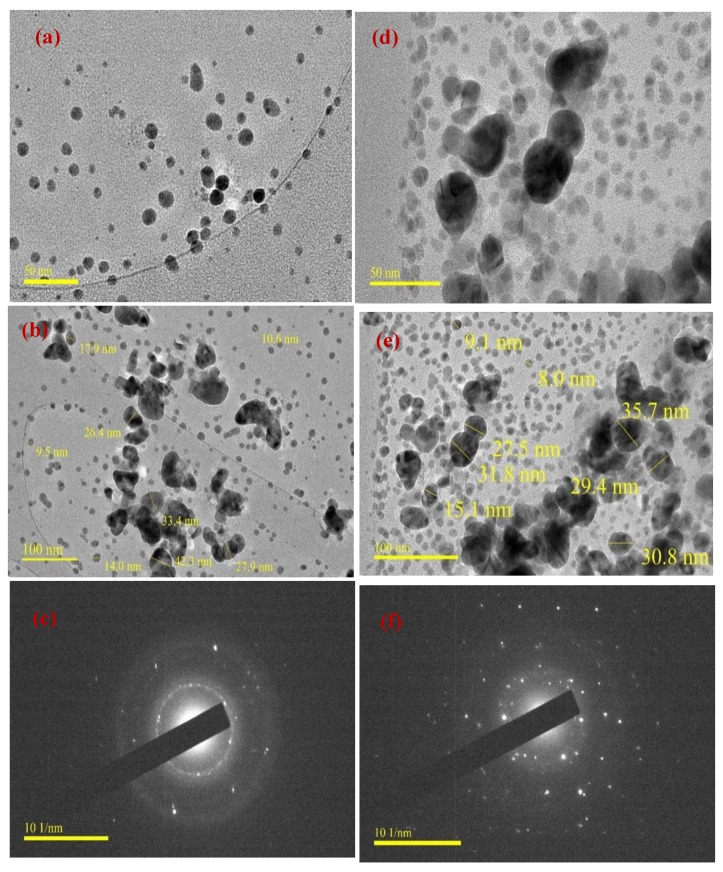
TEM micrographs for (**a**,**b**) DPLE-mediated composite and (**d**,**e**) HEL-mediated composite at (**a**,**d**) 50 nm and (**b**,**e**) 100 nm magnifications; SAED patterns of (**c**) DPLE-mediated composite and (**f**) HEL-mediated composite.

The morphologies and sizes of the biosynthesized AgNPs were determined by TEM analysis. Figure 5 shows TEM images for (a,b) DPLE-mediated composite and (d,e) HEL-mediated composite at (a,d) 50 nm and (b,e) 100 nm magnifications. The selected area electron diffraction (SAED) patterns of DPLE-mediated composite and HEL-mediated composite is given as Figure 5c,f, respectively. The synthesized AgNPs can be clearly seen in Figure 5a–d. The AgNPs are polydispersed, spherical in shape, and of different sizes. The size of AgNPs in DPLE-mediated composite is in the range of 9.5–42.4 nm (Figure 5b) and 8.0–35.7 nm in HEL-mediated composite (Figure 5e). Authors [6,30] had previously linked the diversity in size to differences in formation time. The diffraction rings observed in Figure 5c,f is consistent with the face-centered cubic crystalline (FCC) lattice of silver [24,30]. This further confirms the formation and incorporation of elemental silver in the chitosan backbones. The SAED pattern also indicates that, the AgNPs are polycrystalline [30,43], for instance, the diffraction spots are distributed on concentric circles.

### 3.5. Zeta Potential (ZP) and Polydispersity Index (PDI) Studies

ZP value has been frequently used to define nanoparticles colloid stability [44,45,46]. The categorization is as follows: ZP value in the range ±(0–10) mV is indicative of a highly unstable colloid [44,45]. ZP value of ±(10–20) mV and ±(20–30) mV is reflective of relatively and moderately stable colloid, respectively [44,45]. For highly stable nanoparticles colloid, the ZP value is expected to be greater than ± 30 mV [44,45]. Herein, the ZP obtained for the DPLE- and HEL-mediated composites is +45.6 ± 12.3 mV and +55.6 ± 10.1 mV, respectively. These values indicate that, the synthesized nanofluids are highly stable. The higher ZP value of the HEL-mediated composite relative to the DPLE-mediated composite, again disclose the effect of reducing agent on the reduction and stability of nanoparticles.

The PDI value can be used to articulate the distribution pattern of nanoparticles in a system [47,48]. A PDI value of less than 0.1 infers a highly monodispersed nanoparticles system [47,48]. A PDI value higher than 0.4 is indicative of highly polydispersed nanoparticles system while the PDI value ranging from 0.1 to 0.4 shows a moderately dispersed nanoparticles system [47,48]. The obtained PDI value for DPLE- and HEL-mediated composites is 0.240 and 0.411, respectively. The broadness of the UV-vis peak (Figure 4) can therefore be linked to the observed polydispersion of the nanoparticles.

### 3.6. XRD Studies

**Figure 6:**
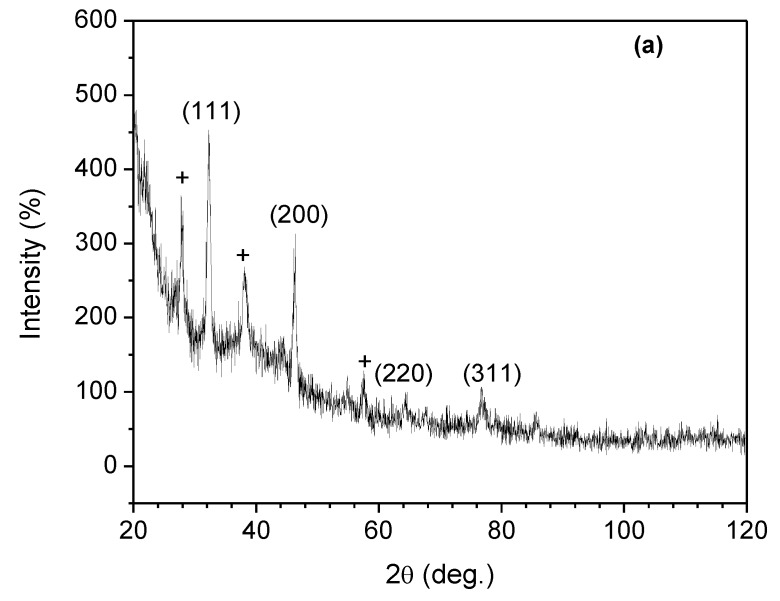

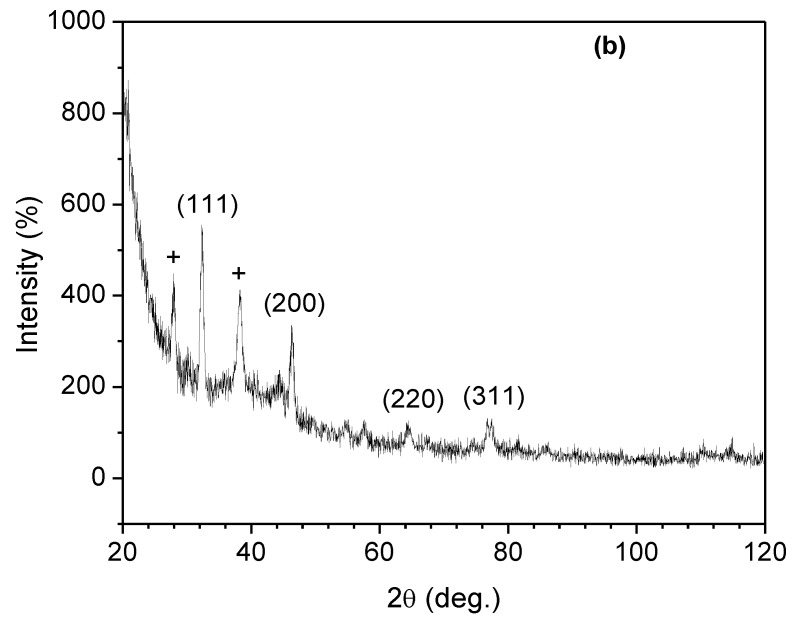
XRD pattern of AgNPs in the composite obtained by treating 5 mL of (**a**) DPLE and (**b**) HEL leaves extracts with 2 g/L chitosan + 0.02 g aqueous AgNO_3_ solution.

The XRD pattern of AgNPs in the composite obtained by treating 5 mL of (a) DPLE and (b) HEL leaves extracts with 2 g/L chitosan + 0.02 g aqueous AgNO_3_ solution is shown in Figure 6. In Figure 6a, the peaks corresponding to the lattice plane of (111), (200), (220), and (311) of a FCC metallic Ag are seen at 2θ = 38.00°, 46.35°, 63.80°, and 76.23°, respectively (JCPDS Card No. 04-0783). Similarly, these peaks are found at 38.56°, 46.33°, 63.33°, and 78.27° in the XRD spectrum of the HEL-mediated composite (Figure 6b). These results further confirm the successful conversion of Ag^+^ to Ag^0^ by the extract. The particle size (*D*) as calculated using the Debye–Scherrer equation (Equation (1)) [7,49] is in the range of 14.00–15.86 nm for DPLE-mediated composite and 8.57–14.62 nm for HEL-mediated composite, which is in agreement with the TEM results (Figure 5b,e). However, beside the assigned FCC peaks, additional peaks (marked with “+” in Figure 5) are observed because the presence of impurities in the synthesized nanofluids [7,26].

The impurities could originate from the mineral elements in the plant extracts that converged on the surface of the nanoparticles [7,45] or from the unconverted silver nitrate.

(1) $$D=\frac{0.9\lambda }{\beta cos\theta }$$

where λ is the wave length of X-ray, *β* is the full width at half maximum (FWHM), and *θ* is the diffraction angle.

### 3.7. Antimicrobial Studies

**Figure 7:**
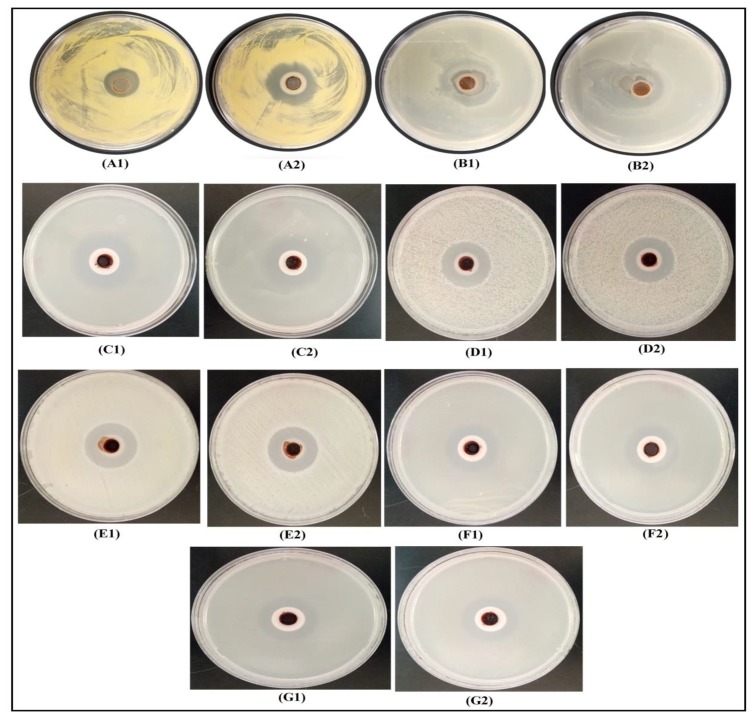
Inhibition of bacterial growth by cup plate experiment. A1, B1, C1, D1, E1, F1, and G1 show inhibition of *P. aeruginosa*, *B. licheniformis*, *E. coli*, *B. cereus*, *P. citronellolis*, *M. luteus*, and *S. haemolyticus* by DPLE-mediated nanocomposite, respectively. A2, B2, C2, D2, E2, F2, and G2 show the inhibition of *P. aeruginosa*, *B. licheniformis*, *E. coli*, *B. cereus*, *P. citronellolis*, *M. luteus*, and *S. haemolyticus* by HEL-mediated nanocomposite, respectively.

**Figure 8:**
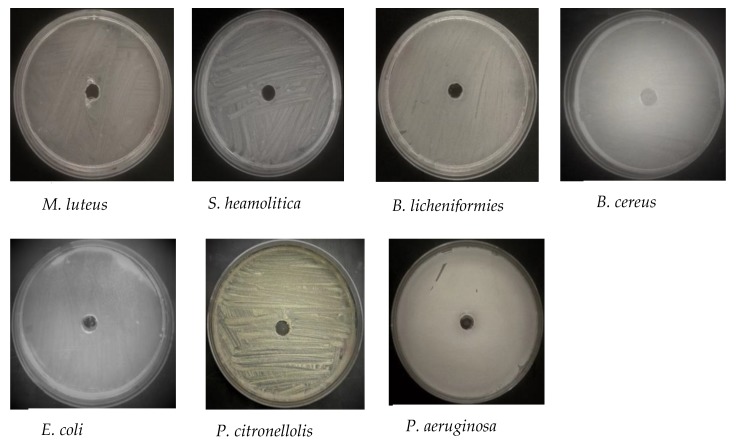
Cup plate experiments showing the negative effect of water against studied bacteria.

The antimicrobial characteristics of the synthesized DPLE- and HEL-mediated composites against pathogenic Gram-positive (*B. licheniformis*, *S. haemolyticus*, *B. cereus*, and *M. luteus*) and Gram-negative (*P. aeruginosa*, *P. citronellolis*, and *E. coli*) bacteria were studied using cup-plate technique (Figure 7). To show that the water used for the preparation of the nanofluids did not contribute to the antimicrobial activity of the nanofluids, cup plate experiments were performed with only water (Figure 8). Clearly, water did not inhibit the growth of the studied bacteria. In Figure 7, the ring-like zone observed indicates the bacterial growth inhibition by the synthesized nanofluids. The distance of the inhibition zone is given in Table 1. The inhibition zone is in the range of 7.0–11.0 mm for DPLE-mediated composite and 7.5–14.0 mm for HEL-mediated composite, except for *S. haemolyticus*. According to the SNV 195920-1992 Standard Antibacterial test, inhibition zone higher than 1 mm is indicative of good antimicrobial potential [50,51]. Thus, these data show that both DPLE- and HEL-mediated composites are very active against the studied microorganisms, which is in line with past reports on the antimicrobial activity of natural polymers/silver nanocomposites [52,53]. Overall, the data show that, HEL-mediated composite is more active than the DPLE-mediated composite. This may be due to smaller size of nanoparticles in HEL-mediated composite than in DPLE-mediated composite (Figure 5), which is in line with previous reports that smaller size AgNPs are more active than larger size nanoparticles [50,51,54]. The smaller size nanoparticles are proffered to readily attach to the cell membrane, penetrate inside the cell, and accumulate in the bacteria more than the bigger ones can do.

To further gain insight into the antibacterial activities of HEL- and DPLE-mediated composites, the bacterial inhibition growth was undertaken in liquid medium. The results show that the dosages of HEL- and DPLE-mediated composites required for the complete inhibition of the tested bacterial strains are 0.1–10% and 1.0–10 %, respectively (Table 2). These data also confirm that, the HEL-mediated composite is more effective than the DPLE-mediated composite.

The higher activity of the HEL-mediated composite over DPLE-mediated composite is also confirmed by the MIC values (Table 3). Indeed, for both Gram-positive and-negative bacteria, the MIC values for DPLE-mediated composite are 1% while those for HEL-mediated composite are 0.1%, except in *P. citronellis* and *B. lecheniformis*. Additionally, the computed MBC values, which reflect the viability of bacteria associated with turbidity, show no difference with those of MIC (Table 3). Thus, the turbidity of the cultures was mainly associated with viable bacteria.

It is hypothesized that silver nanoparticles (AgNPs) inhibit microorganism growth through one or all of the following ways [50,51,54]. Firstly, they can penetrate microorganism membrane and interact with the interior components such that the cells viability is affected. Secondly, they can interact with the sulfhydryl or disulfide groups of microorganisms’ DNA and enzymes and prevent their replication, causing cells death. Thirdly, because they possess surface positive charge, they can attach to the negatively charged bacteria cell membrane, disrupt the cell wall, and destroy the cells. The strong antimicrobial activity observed for the synthesized nanofluids may be due to the release of AgNPs that attack the bacteria through any or all of the above mechanisms. Additionally, low molecular weight chitosan had been reported to exhibits antimicrobial. Costa et al. [55] explained that the effect is due to the capability of chitosan to interact and damage the cell wall of microorganisms through pore formation or membrane disruption. Therefore, the outstanding antimicrobial activity of DPLE- and HEL-mediated composites could be a combined effect of chitosan and AgNPs.

### 3.8. A Comparison of the Antimicrobial Performance of DPLE- amd HEL-mediated Composites with the Individual Components (HEL, DPLE Extracts, AgNPs, and Chitosan Solution)

As earlier mentioned, AgNPs [7] and chitosan [55] had been reported to exhibit antimicrobial effect. To determine whether or not there is combined antimicrobial effect, the performance of HEL- and DPLE-mediated composites was compared with the antimicrobial effect of the individual components used in the synthesis of the composites. The antimicrobial performance of the synthesized chitosan/AgNPs nanofluids, HEL and DPLE extracts, AgNPs, and chitosan solution is given in Table 4. As expected, the plant extracts (HEL and DPLE) exhibited no inhibitory effect towards most of the tested bacteria. HEL extract is however found to minimally inhibit the growth of *S. haemolyticus* and *B. licheniformis* with inhibition zone of 5.0 ± 1.0 mm and 1.0 ± 1.0 mm, respectively. In agreement with previous reports, AgNPs and chitosan exhibited antimicrobial activity against all the tested bacteria. A comparison of the performance of AgNPs with that of chitosan reveals that chitosan is a better antimicrobial agent than AgNPs. For instance, the inhibition zone of chitosan for all the studied microorganisms is larger than that of AgNPs. As explained earlier, chitosan interacts and damages the cell wall of bacteria through pore formation and/or membrane disruption [55]. By comparing the performance of AgNPs and chitosan with those of HEL- and DPLE-mediated composites, it is clear that the nanofluids performance is superior. This could be ascribed to the combined effect of all the components.

## 4. Conclusions

In this article, the synthesis, characterization, and antimicrobial effect of chitosan/silver nanocomposites prepared using *Phoenix dactylifera* (DPLE) or *Rumex vesicarius* (HEL) extracts as the reducing agent is reported. The successfully preparation of the nanofluids was confirmed using FTIR, UV-vis, XRD, and TEM. The silver nanoparticles are polydispersed in the chitosan matrix according to the polydispersity index values and are highly stable. The zeta potential of the silver nanoparticles in DPLE- and HEL-mediated composites is + 46 mV and +56 mV, respectively. The nanoparticles are stabilized in the polymer matrix through the free carboxylate groups in the plant extract according to the FTIR results. HEL is a better reducing agent than DPLE and produced smaller nanoparticles (8.0–36 nm) than DPLE (10–43 nm). DPLE- and HEL-mediated composites effectively inhibit the growth of *P. aeruginosa*, *P. citronellolis, E. coli*, *B. licheniformis*, *B. cereus*, and *M. luteus* but are less active against *S. haemolytic*. However, HEL-mediated composite exhibits a higher antimicrobial effect than DPLE-mediated composite. The higher antimicrobial activity of HEL-mediated composite is linked to the smaller nanoparticles.

## Figures and Tables

**Table 1:** Antimicrobial effect of DPLE- and HEL-mediated nanocomposite using the cup plate experiments. Values represent the diameter (in mm) of the inhibition zone. Data are expressed as mean + standard deviation of duplicate samples.

Type	Bacterium	Diameter of Inhibition (in mm)	
		DPLE-Mediated Composite	HEL-Mediated Composite
Gram negative	*P. aeruginosa*	10.0 ± 2.0	12.0 ± 1.7
	*P. citronellolis*	9.0 ± 1.4	11.0 ± 1.4
	*E. coli*	10.0 ± 2.8	12.5 ± 0.7
Gram positive	*B. licheniformis*	7.0 ± 1.4	7.5 ± 0.7
	*S. haemolyticus*	6.0 ± 1.4	6.5 ± 1.4
	*B. cereus*	11.0 ± 1.4	10.0 ± 0.0
	*M. luteus*	10.0 ± 0.0	14.0 ± 1.4

**Table 2:** Assessment of the antimicrobial effect of DPLE- and HEL-mediated nanocomposites on seven bacteria based on turbidity visualization of the culture and bacterial count on solid Agar plate. Values in brackets represent bacteria counts or colony forming units (CFU × 10^6^/ml) after 24 h culture. Positive control presents culture liquid without bacterial inhibitor substance, and negative control is the culture without bacteria. ‘–’ infers complete growth inhibition where ‘+’ indicate bacteria growth.

Bacterium	DPLE-Bionanofluid (%)					HEL-Bionanofluid (%)					Controls	
	10	1	0.1	0.01	0.001	10	1	0.1	0.01	0.001	+ve	-ve
*P. aeruginosa*	- (0)	- (0)	+ (0.5)	++ (0.8)	+++ (184)	- (0)	- (0)	- (0)	+ (0.36)	+++ (1.1)	++++ (6800)	- (0)
*B. licheniformis*	- (0)	- (0)	+ (0.06)	++ (128)	+++ (13600)	- (0)	- (0)	- (0)	+ (84)	++ (520)	++++ (17000)	- (0)
*E. coli*	- (0)	- (0)	+ (0.7)	++ (1.4)	+++ (7400)	- (0)	- (0)	- (0)	+ (0.6)	++ (5600)	++++ (19000)	- (0)
*B. licheniformis*	- (0)	- (0)	+ (0.006)	++ (128)	+++ (13600)	- (0)	- (0)	+ (0)	+ (84)	++ (520)	++++ (17000)	- (0)
*S. haemolyticus*	- (0)	- (0)	+ (0.03)	++ (72)	+++ (68)	- (0)	- (0)	+ (0.34)	+ (80)	++ (94)	++++ (820000)	- (0)
*B. substilis*	- (0)	- (0)	+ (0.009)	++ (0.18)	+++ (38)	- (0)	- (0)	- (0.11)	+ (1.78)	++ (44)	++++ (23600)	- (0)
*M. aloeverae*	- (0)	- (0)	+ (0.0018)	++ (1.7)	+++ (8000)	- (0)	- (0)	- (0)	+ (3.8)	++ (11000)	++++ (36000)	- (0)

**Table 3:** Minimum inhibitory concentration that inhibit bacteria growth based on visual turbidity or bacteriostatic effect (MIC, in %), and minimum inhibitory concentration that inhibit growth in solid plate culture or bactericidal effect (MBC, in %).

Bacterium	DPLE-Mediated Composite (%)		HEL-Mediated Composite (%)	
	MIC	MBC	MIC	MBC
*P. aeruginosa*	1.0	1.0	0.1	0.1
*P. citronellolis*	1.0	1.0	1.0	1.0
*E. coli*	1.0	1.0	0.1	0.1
*B. licheniformis*	1.0	1.0	1.0	1.0
*S. haemolyticus*	1.0	1.0	0.1	0.1
*B. cereus*	1.0	1.0	0.1	0.1
*M. luteus*	1.0	1.0	0.1	0.1

**Table 4:** Comparative antimicrobial effect of DPLE- and HEL-mediated composite with the synthesis components using the cup plate experiments. Values represent the diameter (in mm) of the inhibition zone.

Type	Bacterium	Diameter of Inhibition (in mm)					
		DPLE-Mediated Composite	HEL-Mediated Composite	DPLE Extract	HEL Extract	AgNPs	Chitosan Solution
Gram negative	*P. aeruginosa*	10.0 ± 2.0	12.0 ± 1.7	0.0 ± 0.0	0.0 ± 0.0	5.0 ± 1.4	8.0 ± 1.4
	*P. citronellolis*	9.0 ± 1.4	11.0 ± 1.4	1.0 ± 0.0	0.0 ± 0.0	5.0 ± 1.0	7.0 ± 1.4
	*E. coli*	10.0 ± 2.8	12.5 ± 0.7	0.0± 0.0	0.0 ± 0.0	4.0 ± 1.0	10.0 ± 2.0
Gram positive	*B. licheniformis*	7.0 ± 1.4	7.5 ± 0.7	0.0 ± 0.0	1.0 ± 0.0	2.0 ± 0.0	8.0 ± 0.0
	*S. haemolyticus*	6.0 ± 1.4	6.5 ± 1.4	0.0 ± 0.0	5.0 ± 1.0	2.0 ± 0.5	6.0 ± 1.0
	*B. cereus*	11.0 ± 1.4	10.0 ± 0.0	0.0 ±0.0	0.0 ± 0.0	4.0 ± 1.5	8.0 ± 1.4
	*M. luteus*	10.0 ± 0.0	14.0 ± 1.4	0.0 ± 0.0	0.0 ± 0.0	2.0 ± 0.0	12.0 ± 2.8
